# Investigation of Growth and Ginsenoside Content of Wild-Simulated Ginseng Cultivated in Different Vegetation Environments for Establishing a Plant Growth Model

**DOI:** 10.3390/plants14060906

**Published:** 2025-03-14

**Authors:** Yeong-Bae Yun, Myeongbin Park, Yi Lee, Yurry Um

**Affiliations:** 1 Forest Medicinal Resources Research Center, National Institute of Forest Science, Yeongju 36040, Republic of Korea; yybangel@korea.kr (Y.-B.Y.); parkmbin@korean.kr (M.P.); 2 Department of Industrial Plant Science and Technology, Chungbuk National University, Cheongju 28644, Republic of Korea; leeyi22@hanmail.net

**Keywords:** ginsenoside, growth characteristics, plant growth model, soil physicochemical properties, wild-simulated ginseng

## Abstract

Wild-simulated ginseng (WSG, *Panax ginseng* C.A. Meyer) is one of the most valuable medicinal plants in the world. This study aimed to investigate the correlation between growth and ginsenoside content of WSG in two different cultivation environments: coniferous and mixed forests. The results showed that air temperature, soil moisture content, and solar radiation were higher in mixed forest than in coniferous forest. Regarding soil properties, electrical conductivity, organic matter, total nitrogen, exchangeable potassium, and magnesium were higher in mixed forest than in coniferous forest. However, exchangeable sodium was lower in mixed forest than in coniferous forest. The analysis of growth characteristics revealed that the number of leaflets was significantly higher in WSG cultivated in mixed forest than in WSG cultivated in coniferous forest, whereas rhizome length, root diameter, root weight, and dry weight were significantly higher in coniferous forest. In contrast, total ginsenoside content and the content of each ginsenoside were much higher in WSG cultivated in mixed forest than in WSG cultivated in coniferous forest. The growth of WSG showed significantly positive correlations with electrical conductivity, organic matter, total nitrogen, exchangeable cations (K^+^, Mg^2+^, Na^+^), and cation exchange capacity. The number of leaflets per stem showed significantly positive correlations with six ginsenosides, whereas petiole length showed significantly negative correlations with mRb1, mRc, and Rb1. In conclusion, growth characteristics of WSG were higher in coniferous forest, but ginsenoside contents were higher in mixed forest. These results might be helpful for establishing the most optimal growth model of WSG, which is affected by various environmental factors.

## 1. Introduction

According to the Korea Forest Service, 64% of Korea’s land area is composed of forests [1]. Coniferous trees are the most prevalent, with broad-leaved forests accounting for 26%, mixed forests accounting for 30.5%, and coniferous forests accounting for 43.5% [2]. For efficient management of diverse forest vegetation environments, it is necessary to establish a comprehensive system of various climatic factors (such as air temperature, soil temperature, solar radiation, soil moisture content, and relative humidity) and changes in soil heterogeneity [3]. In particular, the growth and the content of active components of various wild plants grown in natural mountain environments were affected by climatic factors. The plants respond to environmental conditions by the synthesis of several secondary metabolites or new molecules endowed with important physiological and biological activities and constitute the means of plant defense and adaptation [4,5]. Soil properties are highly influenced by the surrounding vegetation, which is already established. They can be significantly altered by dominant tree species, biotic and abiotic activities in the soil, and site differences [6,7]. Environmental factors known to affect plant growth and active ingredients content can be divided into biotic and abiotic factors [6]. Biotic factors mainly include microorganisms living in the rhizospheric soil, such as plant growth promoting rhizobacteria (PGPR) and endophytes [8,9,10,11,12,13,14]. Abiotic factors include wind, temperature, light, soil minerals, salicylic acid, and phytohormones [15,16,17,18,19,20,21]. Therefore, a multifaceted analysis of these influences is required to determine the optimal plant growth model.

Wild-simulated ginseng (WSG, *Panax ginseng* C.A. Meyer) belongs to the Panax genus of the Araliaceae family. It is defined as “a ginseng that is naturally grown in mountainous areas without the installation of artificial facilities by sowing or transplanting seeds or seedlings”. It is currently designated and managed by the Korea Forest Service as the only specially managed forest product [22]. Suitable conditions for growing Korean WSG are known to include a coverage from 80% to 90%, a slope from 15 to 30°, a slightly acidic soil with a soil pH level of 5.5, a north or northeastern slope direction, a diameter at breast height of more than 15 cm, and a tree height of more than 10 m [23]. According to a statistical survey conducted by the Korea Forest Service, the production of WSG increased significantly by 1.9 times (from 130 tons in 2018 to 254 tons in 2023) and the economic value of production increased by about 1.5 times (from 40.8 billion Won in 2018 to 62.8 billion Won in 2023) [24]. The price of WSG increases significantly as the age increases since it requires long-term cultivation of more than 7 years. The fact that the price of WSG increases when the cultivation period increases is due to various factors, including damage and theft by rodents during the cultivation period, labor shortage due to aging, and lack of various sales channels [25]. Therefore, it is necessary to set quality control standards for WSG by presenting scientific data on the differences in growth characteristics and pharmacological component content according to the cultivation environment.

The active ingredients of WSG identified to date can be categorized into saponin type and non-saponin type according to their structural characteristics [26]. The saponin type is represented by ginsenosides, while the non-saponin type includes polyacetylenes, phenolic compounds, acidic polysaccharides, peptides, and amino acid derivatives [27,28]. Ginsenosides were classified into protopanaxadiol and protopanaxatriol based on their structures. They have various pharmacological effects, including anticancer, anti-inflammatory, anti-obesity, and immune-enhancing properties. The composition and content of these ginsenosides may vary depending on various factors, such as cultivation period, cultivation region, meteorological conditions, vegetation environment, and plant parts (aerial, root) [29,30,31,32,33,34,35].

A previous study has compared soil properties and soil microbial communities of WSG experimental sites according to the composition of overstory trees [36]. In contrast to the previous study, this study investigated growth characteristics and ginsenoside contents of WSG grown in experimental sites composed of coniferous and mixed forests. Correlations of growth characteristics with ginsenoside contents of WSG experimental sites and growing environments of WSG experimental sites were also analyzed to identify the influencing factors and optimal growth model conditions for ginseng cultivation.

## 2. Results

### 2.1. Forest Physiognomy and Meteorological Conditions of Wild-Simulated Ginseng Cultivation Sites with Coniferous and Mixed Forests

Topography and forest physiognomy results of WSG experimental sites composed of mixed forest and coniferous forest were shown in Table 1. The mixed forest experimental site had a slope of 15°, a northeastern direction, and an altitude of 735 m. It was mainly composed of broadleaf trees such as *Cornus controversa* Hemsl. ex Prain, *Morus bombycis* Koidz, *Fraxinus rhynchophylla* (Hance) A.E. Murray, and *Populus* × *tomentiglandulosa* T. B. Lee, and coniferous trees such as *Larix kaempferi* (Lamb.) Carriere at a ratio of 6:4. The average height of trees was 11.8 m and the average diameter at breast height was 21.5 cm. On the other hand, the coniferous forest experimental site had a slope of 15°, a northeastern direction, and an altitude of 719 m. There were no broadleaf trees and the site was composed of *L. kaempferi* (Lamb.) Carriere and *Pinus koraienesis* Siebold & Zucc. at a ratio of 9:1. The average height of trees was 14.6 m and the average diameter at breast height was 33.7 cm.

**Figure 1:**
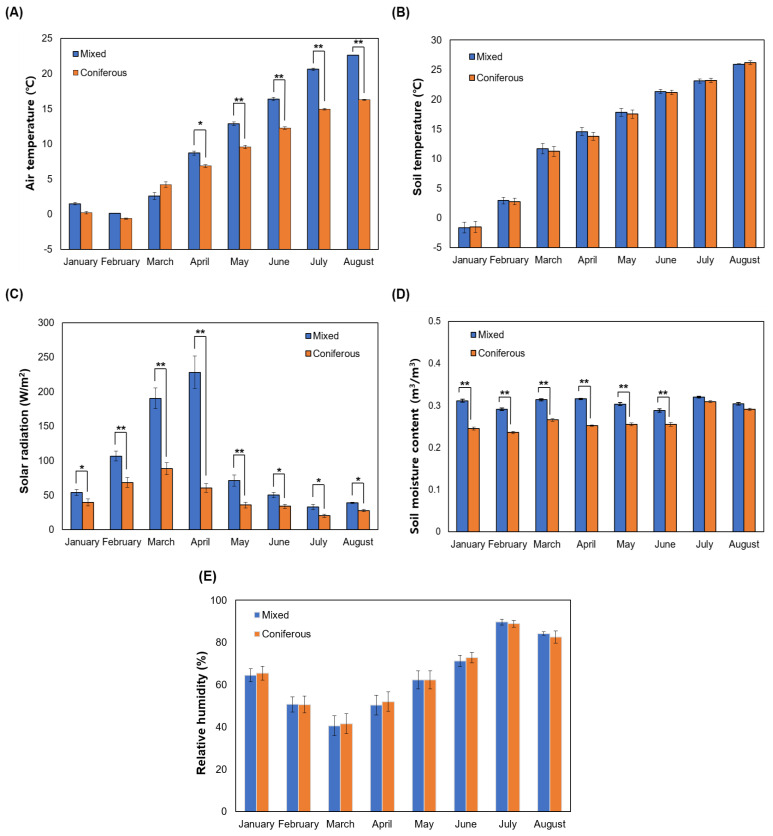
Comparison of the meteorological conditions of wild-simulated ginseng experimental sites consisting of coniferous and mixed forests, respectively. (**A**) Air temperature, (**B**) soil temperature, (**C**) solar radiation, (**D**) soil moisture content, and (**E**) relative humidity. * *p* < 0.05, ** *p* < 0.01.

Measurements of the meteorological environment in the Korean WSG experimental sites composed of mixed forest and coniferous forest showed that air temperature was significantly higher in the mixed forest than in the coniferous forest starting in April (Figure 1A) with soil temperature tending to increase over time from January to August in both sites, although soil temperature showed no significant difference between sites (Figure 1B). Solar radiation was significantly higher in the mixed forest during the entire test period, especially in April when solar radiation was about three times higher in the mixed forest than in the coniferous forest (Figure 1C). The reason for the higher solar radiation in the mixed forest, which was expected to be lower than in the coniferous forest due to the presence of broadleaf trees, might be due to the density of tree species planted in the experimental site where the meteorological equipment was installed. The reason for the sharp decrease in solar radiation in the mixed forest plantation after April has been suggested to be due to the leaf development of broadleaf tree species planted in the mixed forest [36]. The soil moisture content was significantly higher in the mixed forest plantation from January to June. However, in July and August, it was similar in both plantations without showing a significant difference (Figure 1D). Relative humidity was the lowest in March but the highest in July, showing no significant difference between the two plantations (Figure 1E).

### 2.2. Soil Chemical Properties of Wild-Simulated Ginseng Cultivation Sites

A comparison of soil properties of Korean WSG experimental sites composed of mixed forest and coniferous forest showed that the soil pH was acidic at 4.7~4.8 (Table 2), with an electrical conductivity (EC) of 0.3~0.4 dS m^−1^, an organic matter content (OM) of 10.2~14.2%, a total nitrogen content (TN) of 0.4~0.5%, an available phosphorus (Avail. P_2_O_5_) level of 220.3~221.9 mg kg^−1^, an exchangeable potassium (Ex. K^+^) level of 0.1~0.2 cmol^+^ kg^−1^, an exchangeable calcium (Ex. Ca^2+^) level of 2.0~2.9 cmol^+^ kg^−1^, an exchangeable magnesium (Ex. Mg^2+^) level of 0.4~0.7 cmol^+^ kg^−1^, an exchangeable sodium (Ex. Na^+^) level of 0.05~0.1 cmol^+^ kg^−1^, and a cation exchange capacity (CEC) of 25.4~28.3 cmol^+^ kg^−1^. The electrical conductivity (*p* < 0.0152), organic matter content (*p* < 0.0261), total nitrogen content (*p* < 0.0351), exchangeable potassium (*p* < 0.0022), and magnesium (*p* < 0.0162) in the mixed forest experimental site were significantly higher than those in the coniferous forest experimental site. Conversely, the content of exchangeable sodium (*p* < 0.0187) was significantly higher in the soil of coniferous forest experimental site than in the soil of mixed forest experimental site. The soil pH of Korean WSG plantations is acidic or slightly acidic, ranging from 4.0 to 6.0. Soil pH values of the two plantations were not significantly different from this range.

### 2.3. Growth Characteristics of Wild-Simulated Ginseng

**Figure 2:**
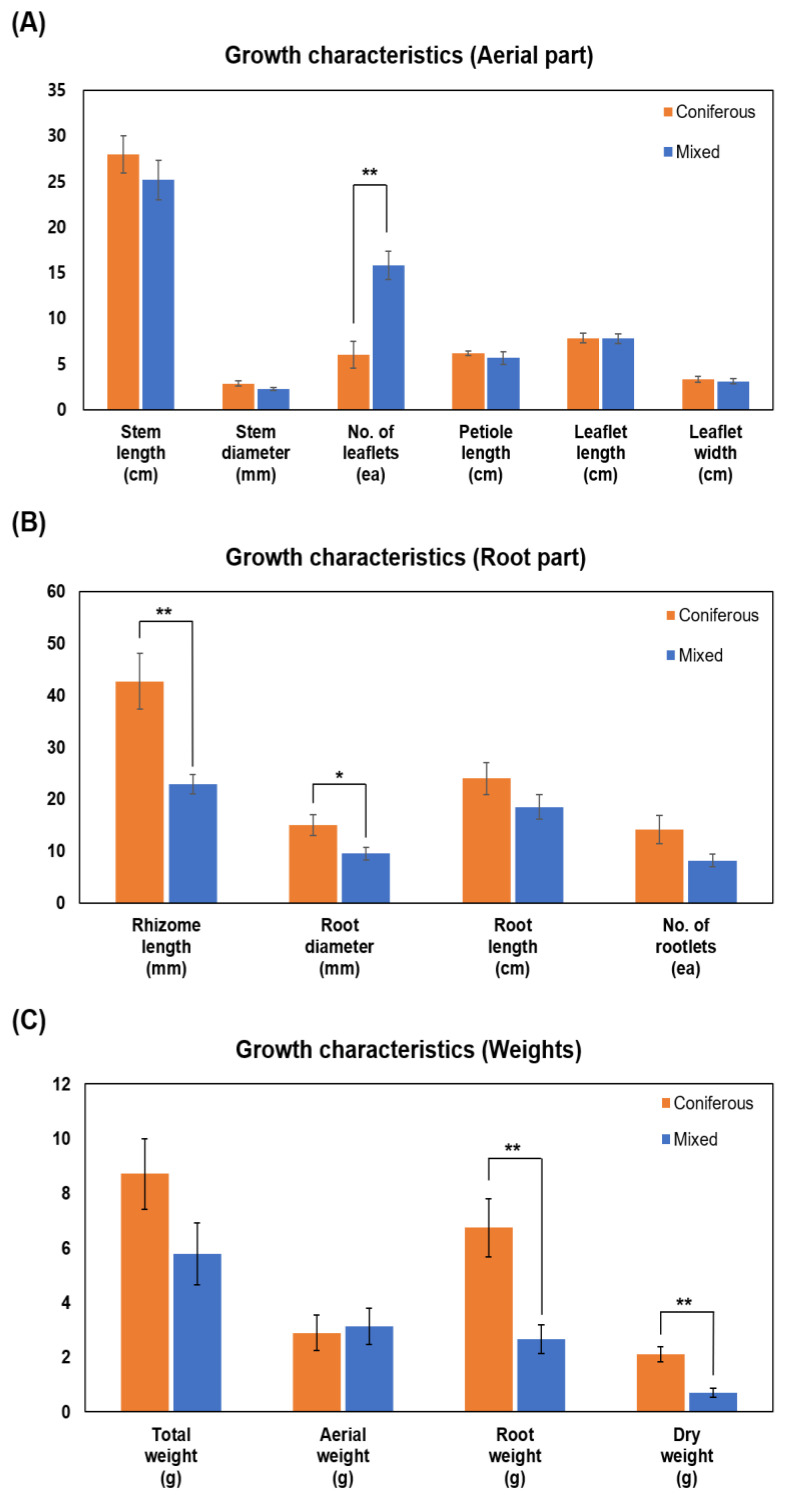
Growth characteristics of wild-simulated ginseng cultivated in coniferous and mixed forests. (**A**) Aerial parts, (**B**) root parts, and (**C**) weights. * *p* < 0.05, ** *p* < 0.01.

In the analysis of growth characteristics of WSG, aerial parts of WSG ranged from 25.2 to 28.0 cm in stem length, from 2.2 to 2.9 mm in stem diameter, from 6 to 15.8 in the number of leaflets per stem, from 5.6 to 6.2 cm in petiole length, 7.8 cm in leaflet length, and ranged from 3.1 to 3.3 cm in leaflet width (Figure 2A). Regarding root parts, the rhizome length ranged from 22.9 mm to 42.8 mm, root diameter ranged from 9.5 mm to 15 mm, root length ranged from 18.5 to 24 cm, and the number of rootlets ranged from 8.2 to 14.2 (Figure 2B). The weight ranged from 5.8 to 8.7 g for total weight, from 2.9 to 3.1 g for the aerial part, from 2.7 to 6.7 g for the root part, and from 0.7 to 2.1 g for dry weight (Figure 2C). For the aerial part, the number of leaflets per stem was significantly higher in WSG cultivated in mixed forest than in WSG cultivated in coniferous forest (*p* < 0.0027). However, root parts and weights were higher in coniferous forests, especially rhizome length (*p* < 0.0086), root diameter (*p* < 0.0499), root weight (*p* < 0.0088), and dry weight (*p* < 0.0026), which showed significant differences (Figure 2B,C).

### 2.4. Ginsenoside Content of Wild-Simulated Ginseng

**Figure 3:**
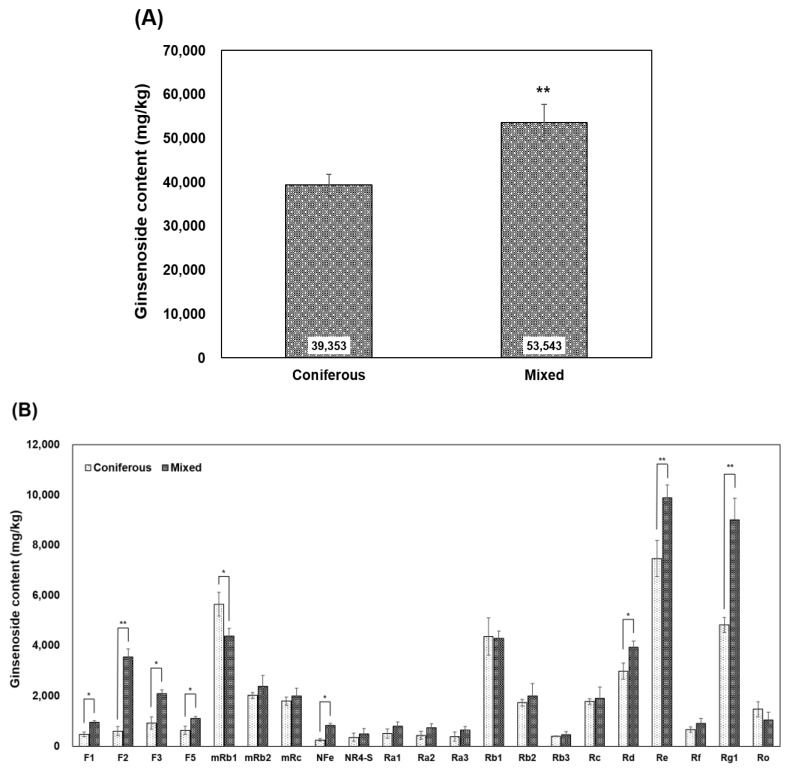
Ginsenoside content of wild-simulated ginseng cultivated in coniferous and mixed forests. (**A**) Total ginsenoside content, (**B**) content of each ginsenoside of wild-simulated ginseng. * *p* < 0.05, ** *p* < 0.01.

When contents of 21 ginsenosides in WSG collected from experimental sites composed of different dominant tree species were added together, the content was about 39.4 g kg^−1^ in WSG collected from the experimental site in coniferous forest and about 53.5 g kg^−1^ in WSG collected from the experimental site in mixed forest. The analysis showed that the total content of ginsenosides was about 1.3 times higher in WSG cultivated in the coniferous forest than in WSG cultivated in the mixed forest *(p =* 0.0098) (Figure 3A).

Regarding ginsenoside compositions, their contents were as follows: [F1] 0.48~0.95 g kg^−1^, [F2] 0.6~3.54 g kg^−1^, [F3] 0.92~2.09 g kg^−1^, [F5] 0.64~1.11 g kg^−1^, [mRb1] 4.38~5.65 g kg^−1^, [mRb2] 2.03~2.39 g kg^−1^, [mRc] 1.8~2.0 g kg^−1^, [NFe] 0.25~0.83 g kg^−1^, [NR4-S] 0.36~0.49 g kg^−1^, [Ra1] 0.5~0.8 g kg^−1^, [Ra2] 0.43~0.74 g kg^−1^, [Ra3] 0.38~0.64 g kg^−1^, [Rb1] 4.28~4.37 g kg^−1^, [Rb2] 1.73~2.01 g kg^−1^, [Rb3] 0.4~0.46 g kg^−1^, [Rc] 1.78~1.91 g kg^−1^, [Rd] 2.99~3.94 g kg^−1^, [Re] 7.47~7.89 g kg^−1^, [Rf] 0.66~0.91 g kg^−1^, [Rg1] 4.82~9.01 g kg^−1^, and [Ro] 1.04–1.48 g kg^−1^. Eighteen out of 21 ginsenosides had higher contents in mixed forest than in coniferous forest (Figure 3B), except for mRb1 (*p* < 0.0447), Rb1, and Ro. In particular, F1 (*p* < 0.0412), F2 (*p* < 0.0068), F3 (*p* < 0.0382), F5 (*p* < 0.0458), NFe (*p* < 0.0439), Rd (*p* < 0.0425), Re (*p* < 0.0088), and Rg1 (*p* < 0.0038) were significantly higher in mixed forest than in coniferous forest.

### 2.5. Correlation Between Soil Chemical Properties, Growth Characteristics, and Ginsenoside Content of Wild-Simulated Ginseng

**Figure 4:**
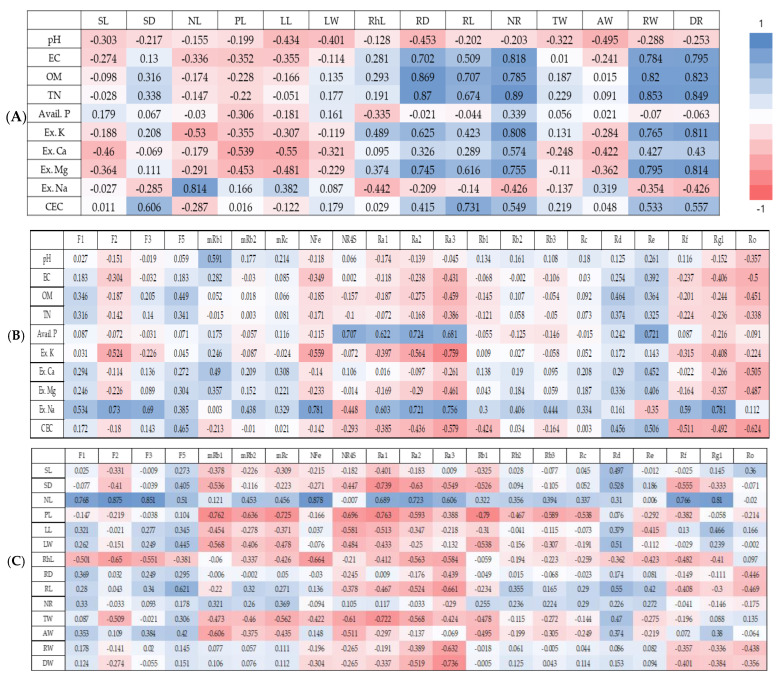
Correlation analysis between soil properties, growth characteristics, and ginsenoside content of wild-simulated ginseng. (**A**) Soil properties–growth characteristics, (**B**) soil properties–ginsenoside content, (**C**) growth characteristics–ginsenoside content. SL: stem length; SD: stem diameter; NL: number of leaflets; PL: petiole length; LL: leaflet length; LW: leaflet width; RhiL: rhizome length; RD: root diameter; RL: root length; NR: number of rootlets; TW: total weight; AW: aerial weight; RW: root weight; DW: dry weight; EC: electrical conductivity; OM: organic matter; TN: total nitrogen; Avail. P: available phosphate; Ex. K: exchangeable potassium; Ex. Ca: exchangeable calcium; Ex. Mg: exchangeable magnesium; Ex. Na: exchangeable sodium; CEC: cation exchange capacity.

When correlations of soil chemical properties of WSG experimental sites with growth characteristics of WSG were analyzed, organic matter content and total nitrogen content were found to be significantly correlated with five growth characteristics (root diameter, root length, number of rootlets, root weight, and dry weight), while electrical conductivity and exchangeable magnesium were significantly correlated with four growth characteristics (root diameter, number of rootlets, root weight, and dry weight) (Figure 4A, Table S1). Exchangeable potassium was significantly correlated with the number of rootlets, root weight, and dry weight. Exchangeable sodium was significantly correlated with only an aerial growth characteristic, which is the number of leaflets per stem. The cationic exchange capacity was significantly correlated with root length.

When the correlation between soil chemical properties of WSG experimental sites and ginsenoside content in WSG was analyzed, the content of exchangeable sodium was found to show a significant positive correlation with contents of ginsenosides F3, NFe, and Rg1. The content of free phosphoric acid also showed a significant positive correlation with ginsenoside Re (Figure 4B, Table S2).

## 3. Discussion

### 3.1. Differences in Topography, Forest Physiognomy, and Soil Chemical Properties of Wild-Simulated Ginseng Experimental Sites Composed of Coniferous and Mixed Forests

A previous study [37] has reported that a slope terrain (vs. flat land), a slope direction of north or northeast, a slope degree of 5°~15°, and a tree height of more than 7 m are optimal for cultivating WSG. The location environment of Korean WSG cultivation sites reported by the Korea Forestry Promotion Agency varies from 5° to 29°, with a slope direction of northeast, northwest, southwest, or southeast; an average diameter at breast height of 21.5 cm; and an average tree height of 16.7 m [20,38,39]. These results are similar to those of mixed and coniferous forests in this study.

Kim et al. [40] have reported that areas with a large number of broadleaf forests are mainly topographically wet with high precipitation, which would favor the growth of moisture-related plants. Compared to coniferous forests, broadleaf forests and mixed forests have been reported to have lower solar radiation due to their high degrees of enclosure, and thus higher relative humidity [41,42]. However, in the present study, although solar radiation was higher in mixed forests, relative humidity was not significantly different between coniferous forests and mixed forests, suggesting that factors that could affect the siting environment of plantations might be influenced not only by solar radiation, but also by wind speed and air pressure [43].

Soils have been reported to be sandy loam, loamy sand, and loamy soil with good drainage [37]. Previous studies have also shown that soils of Korean WSG cultivation sites have low soil pH with high organic matter, total nitrogen content, and cation exchange capacity [34], suggesting that areas composed of mixed forests rather than coniferous forests might be more suitable for growing Korean WSG.

### 3.2. Comparison of Growth Characteristics and Ginsenoside Content of Wild-Simulated Ginseng in Different Forest Vegetation Environments

Kim et al. [36] reported that growth characteristics of WSG can differ depending on the dominant tree species in WSG plantations of different regions, with growth characteristics of WSG collected from plantations composed of mixed forest being significantly higher than those of coniferous forest. The results of the present study differed from those of previous studies as most of the growth characteristics were higher in coniferous forest test plots. The cause of this difference is likely related to the soil microbial community [44] and nutrient uptake [45,46] of the planting site depending on the dominant tree species. Various soil factors not only can inhibit pathogenic microorganisms in the soil, but also can affect the availability of nutrients, which can have a significant impact on plant growth and productivity [47,48,49].

Although most of the growth characteristics of WSG cultivated in mixed forest were inferior to those of WSG cultivated in coniferous forest, contents of ginsenosides were significantly higher in WSG cultivated in mixed forest than in WSG cultivated in coniferous forest. This suggested that coniferous forests are more suitable for growing WSG than mixed forests as an aspect of growth, although mixed forests might be more suitable for growing WSG in terms of pharmacological efficacy. However, the root weight (RW) and dry weight (DW) of WSG collected from coniferous forests were less than half of that of WSG collected from mixed forests before the ginsenoside content was analyzed, suggesting that the same amount of WSG might have a higher content per unit mass. Among the various meteorological conditions measured in this study, solar radiation was significantly higher in the mixed forest WSG experimental site than in the coniferous forest WSG experimental site over the entire period (Figure 1C). Solar radiation plays a crucial role in plant photosynthesis. Thus, it can be hypothesized that the biosynthesis of various bioactive substances, including ginsenosides and active components of WSG, would be more active in mixed forest [50,51,52,53,54,55,56]. The results of ginsenoside content in this study also supported this hypothesis as total contents of 21 ginsenosides and most ginsenosides except for mRb1, Rb1, and Ro were higher in WSG cultivated in mixed forest. The active ingredients of most medicinal crops are secondary metabolites, which are known to play a very important role in mechanisms by which plants can defend themselves in stressful environments [57]. Ginsenosides are representative secondary metabolites of Panax ginseng [58]. They are known to have antioxidant, anti-aging, anticancer, anti-inflammatory, and health-improving activities [59,60]. The composition and content of ginsenosides can vary depending on the growing environment of WSG, with more harsh environments producing more ginsenosides as a defense mechanism for the plant [61,62]. The ginsenoside content of ginseng may also depend on the planting spacing of WSG. Liu et al. [63] have shown that planting at a certain spacing (8 × 8 cm or 10 × 10 cm) can result in a higher ginsenoside content. However, planting quite close together resulted in an accumulation of phytohormones and metabolites related to antioxidants and other stress resistances, suggesting that both the growing environment and planting method are related to the ginsenoside content.

### 3.3. Effects of Soil Chemical Properties, Growth Characteristics, and Ginsenoside Content of Wild-Simulated Ginseng

Since WSG should be cultivated in natural forest soils for a long period of time without using chemically synthesized pesticides or fertilizers, soil properties, soil microbial communities, and the vegetation environment, including the dominant tree species on the site, play a very important role [64,65]. The high organic matter content in the soil is thought to increase the growth of WSG since it allows plants to absorb nitrogen through nitrogen mineralization [66]. In addition, the uptake of potassium is known to promote the uptake of nitrate, since plants take up potassium ion and nitrate simultaneously from the soil by symporter transporter proteins present in plant cell membrane [67,68]. As contents of exchangeable potassium and total nitrogen showed significant positive correlations with the growth of WSG, it could be assumed that higher amounts of nitrogen and potassium in the soil could make it easier for the plant to absorb nitrate and potassium into the plant body and promote plant growth. On the other hand, most of the phosphorus in the soil are in an organic state. Since phosphorus is immobilized through the decomposition process by microorganisms, it is difficult for phosphorus to be used by plants due to its low mobility. This might explain why it does not show a significant correlation with plant growth [69,70].

The community and diversity of plants comprising a plantation can influence the physical and chemical properties of forest soils [71,72]. Previous studies have reported that soil properties differ depending on the proportion of coniferous and mixed forests, with higher proportions of broadleaf species having higher deciduous production than coniferous forests, resulting in higher contents of organic matter and total nitrogen with higher cation exchange capacity [72,73]. Phosphate is an important factor involved in the biosynthetic pathway of ginsenosides, a class of terpenoids. It is used in the synthesis of isopentenyl pyrophosphate, a precursor of triterpenoids [74,75,76,77]. Therefore, the positive correlation between free phosphate content and ginsenoside content may indicate that WSG is able to absorb phosphate from the soil to aid in ginsenoside biosynthesis.

Since the number of leaflets per stem is a growth characteristic that is significantly correlated with ginsenoside content in a previous study of 4-year-old WSG [78], it is possible to predict the ginsenoside content of WSG by measuring the number of leaflets per stem before analyzing the ginsenoside content. In addition, as shown in this study, the growth and ginsenoside content of WSG are not necessarily proportional. The production of ginsenosides of WSG is not significant in an environment suitable for growth [77,79]. Thus, the production of ginsenosides known to be secondary metabolites might have increased for self-defensive pathways of WSG in a harsh cultivation environment, although the growth of WSG might not be significant.

A plant growth model is a virtual simulation of optimal growing conditions by synthesizing climate and soil data and the corresponding aerial and root growth data of a crop. It can predict growth changes in crops that are affected by various environmental factors. The most appropriate crop growth model should be applied considering the purpose and scope of each crop [80]. Plant growth modeling can also predict the potential effects of climate change on crop yields by analyzing responses of crops to given climatic conditions [81,82,83]. In addition, plant root growth and water uptake models can be applied to predict the growth of plant roots in different environments by developing the most appropriate algorithms for nutrient uptake in the soil [84,85,86,87]. Structural and functional aspects of roots must be evaluated in a terrestrial biosphere model [88,89]. The interaction between aerial and root growth of plants must be investigated through mathematical modeling [90]. In particular, since the use of chemicals is legally restricted and ginseng must be cultivated under natural conditions, investigating various environmental factors that might affect the growth and active ingredient biosynthesis of WSG, including climatic conditions (air temperature, solar radiation), soil pH and soil chemical properties (organic matter, exchangeable cations), and soil microbial communities inhabiting the rhizospheric soil of WSG cultivation sites, is essential. In addition, since the law was recently amended to allow the aerial part of WSG to be consumed, it is also necessary to develop a growth modeling algorithm for the interaction between aerial and root parts.

The plant growth model is a method to find the optimal plant cultivation environment by synthesizing various environmental conditions such as plant growth, active components, climate environment, soil physicochemical properties of the planting site, and soil microbial communities. In particular, since the installation of artificial facilities and the treatment of chemicals are limited for wild-simulated ginseng, the environmental conditions of the cultivation site are very important for growth and active components synthesis. Through this study, we analyzed the growth and ginsenoside content of wild-simulated ginseng according to different forest physiognomies and identified the correlation between them to suggest the optimal environmental conditions for wild-simulated ginseng cultivation. Based on the results of this study, the cultivation environment may change for various medicinal crops as the atmospheric temperature and CO_2_ concentration increase due to climate change, and the plant growth model will be able to predict and prepare for changes in the optimal cultivation site according to changes in climate factors. The results of this study showed that the growth and ginsenoside content of WSG could be changed by various environmental factors. If we can develop a growth modeling algorithm for WSG by analyzing the meteorological environment, vegetation environment, soil chemical properties, and soil microbial community known to interact most closely with the plant for WSG plantations composed of various overstory trees in the coniferous and mixed forests investigated in this study, the production of WSG can be increased and conditions of the cultivation site could be determined.

## 4. Materials and Methods

### 4.1. Measurement of Forest Physiognomy and Topography, and Collection of Meteorological Data

Location environmental surveys and meteorological data were collected from January 2023 to August 2023 in coniferous forest (35°54′13.2″ N, 127°50′32.2″ E) and mixed forest (35°54′16.9″ N, 127°50′40.0″ E) plantations, respectively. For the location environmental surveys, 10 m × 10 m plots were selected in the area where plantations were established. Forest physiognomy (tree species, height, diameter at breast height) and topographical characteristics (slope direction, slope degree, height above sea level) of plots were measured. For meteorological data collection, a datalogger (HOBO U30 USB Weather Station, Onset Computer Co., Bourne, MA, USA) was installed in the center of each plot to collect air temperature, soil temperature, solar radiation, soil moisture, and relative humidity data at 1 h intervals.

### 4.2. Collection of Wild-Simulated Ginseng and Rhizospheric Soil Samples

Fourteen-year-old WSG samples were collected from the WSG experimental sites of coniferous and mixed forests in Jeollanbuk-do province of Korea. Five WSG samples were randomly collected from each experimental site (10 m × 10 m). The collected samples were washed with distilled water, and naturally air-dried at room temperature until the surface moisture was removed. After measuring the growth characteristics of the collected WSG samples, all samples were stored at −70 °C. The samples used for the analysis of components were dried in a freeze-dryer, ground using a mortar and pestle, and the powder that passed through an 80-mesh standard sieve was stored at −70 °C prior to use. For soil samples, 100 g of rhizospheric soil was collected after removing the topsoil at a depth from 10 to 30 cm. All soil samples were air-dried in a cool and dry place, filtered through a 2 mm sieve, and stored at room temperature.

### 4.3. Soil Chemical Properties of Wild-Simulated Ginseng Experimental Sites

Soil chemical properties of WSG experimental sites were analyzed in accordance with the Comprehensive Laboratory Analysis Manual published by the Rural Development Administration [91]. Soil pH and electrical conductivity (EC) were determined using a pH meter and an EC meter, respectively, after mixing the soil with distilled water in a 1:5 ratio and agitating for 30 min. The organic matter (OM) content was assessed via the Walkley-Black method, while the total nitrogen (TN) content was measured using the Kjeldahl distillation method after treating 1 g of soil with 5 mL of concentrated sulfuric acid and processing it in a block digester. The available phosphate (Avail. P) content was quantified by absorbance using 1-amino-2-naphtol-sulfanic acid via the Lancaster leaching method. The exchangeable cations (Ex. K^+^, Ex. Ca^2+^, Ex. Mg^2+^, Ex. Na^+^) content was determined through Inductively Coupled Plasma Optical Emission Spectrometry (ICP-OES) after leaching the soil with 1 N-ammonium acetate (NH_4_OAc), and the cation exchange capacity (CEC) was measured by the Kjeldahl distillation of substituted NH_4_^+^ in soil after leaching with 1 N-NH_4_OAc.

### 4.4. Investigation of Growth Characteristics of Wild-Simulated Ginseng

The growth characteristics investigation for the collected WSG was conducted in accordance with the guidelines for the crop-specific characteristics manual (ginseng TG). The aerial parts examined included the stem length (SL), stem diameter (SD), number of leaflets per stem (NL), petiole length (PL), leaflet length (LL), and leaflet width (LW). The root parts were rhizome length (RhL), root diameter (RD), root length (RL), and number of rootlets (NR). Weights were measured as total weight (TW), aerial weight (AW), and root weight (RW) [92]. The length characteristics of aerial and root parts of WSG were measured using a ruler, the diameter (thickness) was measured using a Vernier calipers (FUTURO IP67 Connected, Urdorf, Switzerland), and the weight was measured using chemical balance.

### 4.5. Extraction of Wild-Simulated Ginseng and Reagents

For the analysis of ginsenosides in 14-year-old WSG, 0.2 g of powder sample was added to 10 mL of 70% methanol, followed by ultrasonic extraction (JAC-5020, KODO, Hwaseong, Republic of Korea) for 30 min. The extract was centrifuged in a centrifuge (Labogene, BMS, Seoul, Republic of Korea) for 10 min. The supernatant was filtered through a 0.2 μm membrane filter (Whatman Syringe Filter, Maidstone, UK). The filtrate was diluted 10-fold with distilled water for analysis. The ginsenoside standards used in the analysis were purchased from Chemfaces (Wuhan, China). Methanol, acetonitrile, and the sterile distilled water used in the extraction and HPLC analysis were purchased from J.T. Baker (Easton, PA, USA).

### 4.6. Ginsenoside Content Analysis of Wild-Simulated Ginseng Samples

The quantitation of ginsenosides was conducted by a LC–ESI–MS/MS system (LCMS-8050 system, Shimadzu, Kyoto, Japan) in the negative mode electrospray ionization. The LC condition was as follows: LC separation was on a C18 column (Cortecs^®^UPLC^®^T3 1.6 μm, 2.1 × 150 mm, Waters, Milford, USA) using gradient elution with solvent A (0.1%, *v*/*v*, formic acid in water) and solvent B (0.1%, *v*/*v*, formic acid in acetonitrile including 10% methanol). The gradient elution was conducted as follows: 35% solvent B (0.5 min), 40% solvent B (6.0 min), 45% solvent B (7.0 min), 70% solvent B (14.0 min), 75% solvent B (16.0 min), and 95% solvent B (16.5 min). The elution flow rate was 0.45 mL/min and the sample injection volume was 1.0 μL. The temperature conditions in the mass spectrometer were as follows: interface temperature of 300 °C, desolvation temperature of 250 °C, and heating block of 400 °C. The gas conditions were as follows: nebulizing nitrogen gas of 3.0 mL/min, heating nitrogen gas of 10.0 mL/min, and drying nitrogen gas of 10.0 mL/min. The precursors and product ions of ginsenoside standards in mass spectrometry were determined by an automated process in LC/MS spectrometer, and the MRM (multiple reaction monitoring) conditions of ginsenoside standards were determined such as Q1 pre-bias voltage, dwell time, collision energy, and Q3 pre-bias voltage. Then, these conditions were collected and applied to the ginsenoside analyses of samples. Quantitation of the ginsenoside was conducted by an internal (digoxin) linear regression method to the peak area. Both the information peak area at a unique retention time and product ions information were used for each ginsenoside identification and quantitation.

### 4.7. Statistical Analysis and Correlation Analysis

The analyzed data values were expressed as the means ± standard error (SE). The means of each experimental value were tested by the *t*-test using SAS (Statistical Analysis System ver. 7.1) software at a significance level of 5% (*p* < 0.05). Correlations between soil chemical properties, growth characteristics, and ginsenoside content of WSG were analyzed using IBM SPSS statistics (version 25, IBM Corp., Armonk, NY, USA). Additionally, the Pearson’s correlation coefficient (r) and significance (*p* < 0.05) were obtained.

## 5. Conclusions

In this study, we analyzed the effects of various environmental factors on the growth and active ingredients content of WSG, one of the most famous medicinal crops, which were changed by various environmental factors, and identified the correlations among them. Furthermore, it was found that WSG, where the installation of artificial facilities and the treatment of chemicals are restricted, is affected by various factors such as climatic conditions, meteorological environment, vegetation environment, soil characteristics, and soil microbial community, which can interact most closely with the WSG plantations composed of various upper layer trees. Therefore, the results of this study suggest that a thorough analysis of various climatic conditions and environmental factors should be conducted when developing a crop growth model for WSG. Based on the results of this study, if a growth model for WSG is developed, the production of WSG can increase and determine the optimal conditions of the cultivation site.

## Figures and Tables

**Table 1:** Forest physiognomy and topography of wild-simulated ginseng experimental sites.

Experimental Site	Topography			Forest Physiognomy				
	Slope		Altitude	Species of Tree		TH ^z^	DBH ^y^	Percentage
	Degree	Direction	m			m	cm	%
Mixed (35°54′16.9″ N, 127°50′40.0″ E)	15	East north	735	Broad-leaved	*Cornus controversa* Hemsl. ex Prain	6	7.5	14.3
					*Morus bombycis* Koidz.	12	19	14.3
					*Fraxinus rhynchophylla* (Hance) A.E. Murray	13	20	14.3
					*Populus* × *tomentiglandulosa* T. B. Lee	13	24	14.3
				Coniferous	*Larix kaempferi* (Lamb.) Carriere	15	37	42.8
Coniferous (35°54′13.2″ N, 127°50′32.2″ E)	15	East north	719	Broad-leaved	ND ^x^	ND	ND	ND
				Coniferous	*Pinus koraienesis* Siebold & Zucc.	14	40	10
					*Larix kaempferi* (Lamb.) Carriere	15.1	27.3	90

^z^ TH: tree height; ^y^ DBH: diameter at breast height; ^x^ ND: not determined.

**Table 2:** Soil chemical properties of wild-simulated ginseng experimental sites.

	pH	EC ^z^	OM ^y^	TN ^x^	Avail. P_2_O_5_ ^w^	Ex. K^+ v^	Ex. Ca^2+ u^	Ex. Mg^2+ t^	Ex. Na^+ s^	CEC ^r^
	[1:5]	(dS/m)	(%)		(mg/kg)	cmol^+^/kg				
Mixed	4.7 ± 0.1	0.4 ± 0.0	14.2 ± 1.4	0.5 ± 0.0	220.3 ± 13.9	0.2 ± 0.0	2.9 ± 0.6	0.7 ± 0.1	0.05 ± 0.0	28.3 ± 1.6
Coniferous	4.8 ± 0.1	0.3 ± 0.0	10.2 ± 0.3	0.4 ± 0.0	221.9 ± 28.5	0.1 ± 0.0	2.0 ± 0.5	0.4 ± 0.0	0.1 ± 0.0	25.4 ± 1.3
*p* value	0.1784	0.0234 *	0.0144 *	0.0322 *	0.5384	0.0421 *	0.2712	0.0419 *	0.0371 *	0.2513

Values in each column represent the average of five replicates ± SE. Significant differences according to the t-test at *p* ≤ 0.05 levels are indicated by different letters. Significance is demonstrated as follows: *p* ≤ 0.05 (*). ^z^ EC: electrical conductivity; ^y^ OM: organic matter; ^x^ TN: total nitrogen; ^w^ Avail. P_2_O_5_: available phosphate; ^v^ Ex. K^+^: exchangeable potassium; ^u^ Ex. Ca^2+^: exchangeable calcium; ^t^ Ex. Mg^2+^: exchangeable magnesium; ^s^ Ex. Na^+^: exchangeable sodium; ^r^ CEC: cation exchange capacity.

## Data Availability

The data that support the findings of this study are available from the corresponding author (Y.U.) upon reasonable request.

## Supplementary Materials

The following supporting information can be downloaded at: https://www.mdpi.com/article/10.3390/plants14060906/s1. Table S1: Pearson’s correlation coefficient between soil properties and growth characteristics of wild-simulated ginseng; Table S2: Pearson’s correlation coefficient between soil properties and ginsenoside contents of wild-simulated ginseng; Table S3: Pearson’s correlation coefficient between growth characteristics and ginsenoside contents of wild-simulated ginseng.
