# Supplementary material for: Investigation of Growth and Ginsenoside Content of Wild-Simulated Ginseng Cultivated in Different Vegetation Environments for Establishing a Plant Growth Model
Source: Plants (Basel). 2025 Mar 14;14(6):906. doi: 10.3390/plants14060906 (PMC11944855; doi:10.3390/plants14060906)
Supplement: Supplementary file 1 [file plants-14-00906-s001.zip › plants-3499978-supplementary.pdf]

Table S1. Pearson's correlation coefficient between soil properties and growth characteristics of wild-simulated ginseng

	Stem length	Stem diameter	No. of leaflets	Petiole length	Leaflet length	Leaflet width	Rhizome length	Root diameter	Root length	No. of rootlets	Total weight	Aerial weight	Root weight	Dry weight
pH	-0.303<br>(0.427)	-0.217<br>(0.575)	-0.155<br>(0.691)	-0.199<br>(0.608)	-0.434<br>(0.243)	-0.401<br>(0.284)	-0.128<br>(0.724)	-0.453<br>(0.189)	-0.202<br>(0.576)	-0.203<br>(0.575)	-0.322<br>(0.398)	-0.495<br>(0.175)	-0.288<br>(0.420)	-0.253<br>(0.481)
EC	-0.274<br>(0.476)	0.130<br>(0.738)	-0.336<br>(0.376)	-0.352<br>(0.352)	-0.355<br>(0.349)	-0.114<br>(0.770)	0.281<br>(0.431)	**0.701***<br>**(0.024)**	0.509<br>(0.133)	**0.818****<br>**(0.004)**	0.010<br>(0.981)	-0.241<br>(0.532)	**0.784****<br>**(0.007)**	**0.795****<br>**(0.006)**
OM	-0.098<br>(0.801)	0.316<br>(0.407)	-0.174<br>(0.655)	-0.228<br>(0.555)	-0.166<br>(0.669)	0.135<br>(0.729)	0.293<br>(0.411)	**0.869****<br>**(0.001)**	**0.707***<br>**(0.022)**	**0.785****<br>**(0.007)**	0.187<br>(0.630)	0.015<br>(0.969)	**0.820****<br>**(0.004)**	**0.823****<br>**(0.003)**
TN	-0.028<br>(0.943)	0.338<br>(0.374)	-0.147<br>(0.705)	-0.220<br>(0.569)	-0.051<br>(0.896)	0.177<br>(0.648)	0.191<br>(0.598)	**0.870****<br>**(0.001)**	**0.674***<br>**(0.033)**	**0.890****<br>**(0.001)**	0.229<br>(0.554)	0.091<br>(0.816)	**0.853****<br>**(0.002)**	**0.849****<br>**(0.002)**
Avail. P_2O_5	0.179<br>(0.644)	0.067<br>(0.864)	-0.030<br>(0.938)	-0.306<br>(0.424)	-0.181<br>(0.640)	0.161<br>(0.679)	-0.335<br>(0.344)	-0.021<br>(0.953)	-0.044<br>(0.904)	0.339<br>(0.338)	0.056<br>(0.885)	0.021<br>(0.957)	-0.070<br>(0.847)	-0.063<br>(0.862)
Ex. K	-0.188<br>(0.628)	0.208<br>(0.592)	-0.530<br>(0.142)	-0.355<br>(0.349)	-0.307<br>(0.422)	-0.119<br>(0.761)	0.489<br>(0.152)	0.625<br>(0.053)	0.423<br>(0.223)	**0.808****<br>**(0.005)**	0.131<br>(0.737)	-0.284<br>(0.459)	**0.765****<br>**(0.010)**	**0.811****<br>**(0.004)**
Ex. Ca	-0.460<br>(0.212)	-0.069<br>(0.860)	-0.179<br>(0.645)	-0.539<br>(0.135)	-0.550<br>(0.125)	-0.321<br>(0.400)	0.095<br>(0.795)	0.326<br>(0.358)	0.289<br>(0.418)	0.574<br>(0.083)	0.187<br>(0.521)	-0.422<br>(0.258)	0.427<br>(0.218)	0.430<br>(0.215)
Ex. Mg	-0.364<br>(0.336)	0.111<br>(0.776)	-0.291<br>(0.448)	-0.453<br>(0.221)	-0.481<br>(0.190)	-0.229<br>(0.554)	0.374<br>(0.287)	**0.745***<br>**(0.014)**	0.616<br>(0.058)	**0.755***<br>**(0.012)**	-0.110<br>(0.778)	-0.362<br>(0.339)	**0.795****<br>**(0.006)**	**0.814****<br>**(0.004)**
Ex. Na	-0.027<br>(0.945)	-0.285<br>(0.458)	**0.814****<br>**(0.008)**	0.166<br>(0.669)	0.382<br>(0.310)	0.087<br>(0.823)	-0.442<br>(0.201)	-0.209<br>(0.562)	-0.140<br>(0.700)	-0.426<br>(0.220)	-0.137<br>(0.726)	0.319<br>(0.402)	-0.354<br>(0.315)	-0.426<br>(0.219)
CEC	0.011<br>(0.978)	0.606<br>(0.084)	-0.287<br>(0.454)	0.016<br>(0.968)	-0.122<br>(0.754)	0.179<br>(0.646)	0.029<br>(0.937)	0.415<br>(0.233)	**0.731***<br>**(0.016)**	0.549<br>(0.100)	0.219<br>(0.571)	0.048<br>(0.903)	0.533<br>(0.113)	0.557<br>(0.094)

Correlation coefficient (r) written are significantly correlated between the variables compared. Negative values denote negative correlation and positive values denote positive correlation. Values in brackets means *P* value ($^{**}P<0.01$, $^{*}P<0.05$).

Table S2. Pearson's correlation coefficient between soil properties and ginsenoside contents of wild-simulated ginseng

	F1	F2	F3	F5	mRb1	mRb2	mRc	NFe	NR4S	Ra1	Ra2
pH	0.027<br>(0.945)	-0.151<br>(0.698)	-0.019<br>(0.962)	0.059<br>(0.879)	0.591<br>(0.094)	0.177<br>(0.650)	-0.336<br>(0.342)	-0.434<br>(0.211)	-0.208<br>(0.621)	-0.440<br>(0.323)	-0.139<br>(0.792)
EC	0.183<br>(0.638)	-0.304<br>(0.426)	-0.032<br>(0.935)	0.183<br>(0.637)	0.282<br>(0.462)	-0.030<br>(0.938)	0.092<br>(0.801)	-0.255<br>(0.478)	0.066<br>(0.876)	0.045<br>(0.923)	-0.238<br>(0.650)
OM	0.346<br>(0.361)	-0.187<br>(0.630)	0.205<br>(0.596)	0.449<br>(0.225)	0.052<br>(0.895)	0.018<br>(0.963)	0.261<br>(0.467)	0.030<br>(0.933)	0.032<br>(0.940)	0.105<br>(0.822)	-0.275<br>(0.598)
TN	0.316<br>(0.407)	-0.142<br>(0.716)	0.140<br>(0.720)	0.341<br>(0.370)	-0.015<br>(0.969)	0.003<br>(0.994)	0.264<br>(0.461)	0.036<br>(0.921)	0.078<br>(0.855)	0.187<br>(0.688)	-0.168<br>(0.750)
Avail. P_2O_5	0.087<br>(0.824)	-0.072<br>(0.855)	-0.031<br>(0.937)	0.071<br>(0.857)	0.175<br>(0.653)	-0.057<br>(0.884)	-0.283<br>(0.427)	-0.361<br>(0.305)	0.379<br>(0.354)	0.224<br>(0.628)	0.724<br>(0.103)
Ex. K	0.031<br>(0.936)	-0.524<br>(0.147)	-0.226<br>(0.558)	0.045<br>(0.909)	0.246<br>(0.523)	-0.087<br>(0.825)	-0.046<br>(0.900)	-0.475<br>(0.165)	-0.040<br>(0.925)	-0.214<br>(0.645)	-0.564<br>(0.243)
Ex. Ca	0.294<br>(0.442)	-0.114<br>(0.770)	0.136<br>(0.727)	0.272<br>(0.479)	0.490<br>(0.181)	0.209<br>(0.590)	-0.129<br>(0.723)	-0.354<br>(0.316)	-0.079<br>(0.853)	-0.195<br>(0.675)	-0.097<br>(0.855)
Ex. Mg	0.246<br>(0.523)	-0.226<br>(0.559)	0.089<br>(0.820)	0.304<br>(0.426)	0.357<br>(0.345)	0.152<br>(0.696)	0.297<br>(0.405)	-0.064<br>(0.860)	0.098<br>(0.817)	0.074<br>(0.875)	-0.290<br>(0.577)
Ex. Na	0.534<br>(0.139)	**0.730***<br>**(0.026)**	**0.690***<br>**(0.040)**	0.385<br>(0.306)	0.003<br>(0.994)	0.438<br>(0.239)	0.311<br>(0.382)	**0.699***<br>**(0.024)**	-0.408<br>(0.316)	-0.082<br>(0.862)	0.721<br>(0.106)
CEC	0.172<br>(0.659)	-0.180<br>(0.644)	0.163<br>(0.713)	0.465<br>(0.208)	-0.213<br>(0.582)	-0.010<br>(0.980)	-0.203<br>(0.573)	-0.282<br>(0.430)	-0.368<br>(0.370)	-0.442<br>(0.321)	-0.436<br>(0.387)

Correlation coefficient (r) written are significantly correlated between the variables compared. Negative values denote negative correlation and positive values denote positive correlation. Values in brackets means *P* value (**P<0.01, *P<0.05).

Table S2. (Continued)

	Ra3	Rb1	Rb2	Rb3	Rc	Rd	Re	Rf	Rg1	Ro
pH	-0.045<br>(0.933)	-0.259<br>(0.469)	-0.285<br>(0.425)	0.108<br>(0.782)	0.180<br>(0.643)	0.125<br>(0.748)	0.261<br>(0.497)	0.116<br>(0.767)	-0.152<br>(0.696)	-0.357<br>(0.346)
EC	-0.431<br>(0.394)	-0.029<br>(0.936)	0.028<br>(0.939)	-0.106<br>(0.787)	0.030<br>(0.939)	0.254<br>(0.510)	0.392<br>(0.297)	-0.237<br>(0.540)	-0.406<br>(0.279)	-0.500<br>(0.171)
OM	-0.459<br>(0.359)	0.054<br>(0.883)	0.265<br>(0.459)	-0.054<br>(0.890)	0.092<br>(0.814)	0.464<br>(0.209)	0.364<br>(0.335)	-0.201<br>(0.604)	-0.244<br>(0.526)	-0.451<br>(0.223)
TN	-0.386<br>(0.449)	0.068<br>(0.852)	0.223<br>(0.535)	-0.050<br>(0.898)	0.073<br>(0.852)	0.374<br>(0.321)	0.325<br>(0.393)	-0.224<br>(0.563)	-0.236<br>(0.541)	-0.338<br>(0.373)
Avail. P_2O_5	0.681<br>(0.136)	-0.310<br>(0.383)	-0.385<br>(0.272)	-0.146<br>(0.708)	-0.015<br>(0.970)	0.242<br>(0.531)	**0.721***<br>**(0.028)**	0.087<br>(0.824)	-0.216<br>(0.578)	-0.091<br>(0.816)
Ex. K	-0.759<br>(0.080)	-0.016<br>(0.965)	-0.005<br>(0.990)	-0.058<br>(0.882)	0.052<br>(0.893)	0.172<br>(0.659)	0.143<br>(0.714)	-0.315<br>(0.409)	-0.408<br>(0.276)	-0.224<br>(0.562)
Ex. Ca	-0.261<br>(0.617)	-0.141<br>(0.698)	-0.136<br>(0.707)	0.095<br>(0.807)	0.208<br>(0.591)	0.290<br>(0.449)	0.452<br>(0.222)	-0.022<br>(0.956)	-0.266<br>(0.490)	-0.505<br>(0.165)
Ex. Mg	-0.461<br>(0.357)	0.151<br>(0.678)	0.269<br>(0.452)	0.059<br>(0.881)	0.187<br>(0.630)	0.336<br>(0.377)	0.406<br>(0.279)	-0.164<br>(0.693)	-0.337<br>(0.375)	-0.487<br>(0.183)
Ex. Na	0.756<br>(0.082)	0.314<br>(0.377)	0.390<br>(0.265)	0.444<br>(0.231)	0.334<br>(0.379)	0.161<br>(0.678)	-0.350<br>(0.356)	0.590<br>(0.095)	**0.781***<br>**(0.013)**	0.112<br>(0.774)
CEC	-0.579<br>(0.228)	-0.500<br>(0.141)	-0.160<br>(0.659)	-0.164<br>(0.674)	0.003<br>(0.993)	0.456<br>(0.217)	0.506<br>(0.164)	-0.511<br>(0.160)	-0.492<br>(0.178)	-0.624<br>(0.073)

Correlation coefficient (r) written are significantly correlated between the variables compared. Negative values denote negative correlation and positive values denote positive correlation. Values in brackets means *P* value ($^{**}P<0.01$, $^{*}P<0.05$).

Table S3. Pearson's correlation coefficient between growth characteristics and ginsenoside contents of wild-simulated ginseng

	F1	F2	F3	F5	mRb1	mRb2	mRc	NFe	NR4S	Ra1	Ra2
Stem length	0.025<br>(0.948)	-0.331<br>(0.384)	-0.009<br>(0.982)	0.273<br>(0.476)	-0.378<br>(0.315)	-0.226<br>(0.559)	-0.309<br>(0.418)	-0.215<br>(0.578)	-0.182<br>(0.697)	-0.401<br>(0.431)	-0.183<br>(0.729)
Stem diameter	-0.077<br>(0.844)	-0.410<br>(0.273)	-0.039<br>(0.921)	0.405<br>(0.280)	-0.536<br>(0.137)	-0.116<br>(0.767)	-0.223<br>(0.563)	-0.271<br>(0.480)	-0.447<br>(0.315)	-0.739<br>(0.093)	-0.630<br>(0.180)
No. of leaflets	**0.768***<br>**(0.016)**	**0.875****<br>**(0.002)**	**0.851****<br>**(0.004)**	0.510<br>(0.161)	0.121<br>(0.757)	0.453<br>(0.221)	0.456<br>(0.218)	**0.878****<br>**(0.002)**	-0.007<br>(0.988)	0.689<br>(0.130)	0.723<br>(0.104)
Petiole length	-0.147<br>(0.706)	-0.219<br>(0.571)	-0.038<br>(0.923)	0.104<br>(0.789)	**-0.762***<br>**(0.017)**	-0.636<br>(0.066)	**-0.725***<br>**(0.027)**	-0.166<br>(0.669)	-0.696<br>(0.082)	-0.763<br>(0.078)	-0.593<br>(0.214)
Leaflet length	0.321<br>(0.400)	-0.021<br>(0.956)	0.277<br>(0.470)	0.345<br>(0.364)	-0.454<br>(0.220)	-0.278<br>(0.469)	-0.371<br>(0.326)	0.037<br>(0.924)	-0.581<br>(0.171)	-0.513<br>(0.298)	-0.347<br>(0.501)
Leaflet width	0.262<br>(0.497)	-0.151<br>(0.698)	0.249<br>(0.518)	0.445<br>(0.230)	-0.568<br>(0.111)	-0.406<br>(0.278)	-0.478<br>(0.193)	-0.076<br>(0.846)	-0.484<br>(0.272)	-0.433<br>(0.391)	-0.250<br>(0.632)
Rhizome length	-0.501<br>(0.169)	-0.650<br>(0.058)	-0.551<br>(0.124)	-0.381<br>(0.312)	-0.060<br>(0.877)	-0.337<br>(0.374)	-0.426<br>(0.253)	-0.664<br>(0.051)	-0.210<br>(0.652)	-0.412<br>(0.417)	-0.563<br>(0.245)
Root diameter	0.369<br>(0.328)	0.032<br>(0.935)	0.249<br>(0.519)	0.295<br>(0.442)	-0.006<br>(0.988)	-0.002<br>(0.997)	0.050<br>(0.899)	-0.030<br>(0.939)	-0.245<br>(0.596)	0.009<br>(0.986)	-0.176<br>(0.739)
Root length	0.280<br>(0.465)	0.043<br>(0.912)	0.340<br>(0.370)	0.621<br>(0.074)	-0.220<br>(0.569)	0.320<br>(0.401)	0.271<br>(0.480)	0.136<br>(0.727)	-0.378<br>(0.403)	-0.467<br>(0.351)	-0.524<br>(0.286)
No. of rootlets	0.330<br>(0.387)	-0.033<br>(0.932)	0.093<br>(0.812)	0.178<br>(0.646)	0.569<br>(0.321)	0.401<br>(0.260)	0.480<br>(0.369)	-0.094<br>(0.809)	0.105<br>(0.823)	0.117<br>(0.825)	-0.033<br>(0.951)
Total weight	0.087<br>(0.825)	-0.509<br>(0.162)	-0.021<br>(0.957)	0.306<br>(0.423)	-0.473<br>(0.199)	-0.460<br>(0.213)	-0.562<br>(0.116)	-0.422<br>(0.258)	-0.610<br>(0.146)	-0.722<br>(0.105)	-0.568<br>(0.239)
Aerial weight	0.353<br>(0.352)	0.109<br>(0.780)	0.384<br>(0.308)	0.420<br>(0.261)	-0.606<br>(0.083)	-0.375<br>(0.320)	-0.435<br>(0.242)	0.148<br>(0.704)	-0.511<br>(0.241)	-0.297<br>(0.568)	-0.137<br>(0.795)

Root weight	0.178 (0.646)	-0.141 (0.717)	0.020 (0.959)	0.145 (0.709)	0.077 (0.844)	0.057 (0.885)	0.111 (0.776)	-0.196 (0.612)	-0.265 (0.565)	-0.191 (0.717)	-0.389 (0.446)
Dry weight	0.124 (0.751)	-0.274 (0.475)	-0.055 (0.888)	0.151 (0.697)	0.106 (0.786)	0.076 (0.846)	0.112 (0.775)	-0.304 (0.427)	-0.265 (0.566)	-0.337 (0.513)	-0.519 (0.291)

Correlation coefficient (r) written are significantly correlated between the variables compared. Negative values denote negative correlation and positive values denote positive correlation. Values in brackets means *P* value ($^{**}P<0.01$, $^{*}P<0.05$).

Table S3. (Continued)

	Ra3	Rb1	Rb2	Rb3	Rc	Rd	Re	Rf	Rg1	Ro
Stem length	0.009<br>(0.986)	-0.325<br>(0.394)	0.028<br>(0.943)	-0.077<br>(0.844)	0.045<br>(0.908)	0.497<br>(0.173)	-0.012<br>(0.976)	-0.025<br>(0.948)	0.145<br>(0.709)	0.360<br>(0.341)
Stem diameter	-0.549<br>(0.146)	-0.526<br>(0.146)	0.094<br>(0.810)	-0.105<br>(0.788)	0.052<br>(0.894)	0.528<br>(0.144)	0.186<br>(0.632)	-0.555<br>(0.121)	-0.333<br>(0.381)	-0.071<br>(0.855)
No. of leaflets	0.606<br>(0.202)	0.322<br>(0.398)	0.356<br>(0.346)	0.394<br>(0.294)	0.337<br>(0.375)	0.310<br>(0.416)	0.006<br>(0.989)	**0.766*<br>(0.016)**	**0.810**<br>(0.008)**	-0.020<br>(0.959)
Petiole length	-0.388<br>(0.448)	**-0.790*<br>(0.011)**	-0.467<br>(0.206)	-0.589<br>(0.095)	-0.538<br>(0.135)	0.076<br>(0.846)	-0.292<br>(0.447)	-0.382<br>(0.311)	-0.058<br>(0.882)	-0.214<br>(0.580)
Leaflet length	-0.218<br>(0.679)	-0.310<br>(0.416)	-0.041<br>(0.917)	-0.115<br>(0.768)	-0.073<br>(0.852)	0.379<br>(0.314)	-0.415<br>(0.267)	0.130<br>(0.738)	0.466<br>(0.206)	0.166<br>(0.669)
Leaflet width	-0.132<br>(0.803)	-0.538<br>(0.135)	-0.156<br>(0.688)	-0.307<br>(0.421)	-0.191<br>(0.622)	0.510<br>(0.161)	-0.112<br>(0.775)	-0.029<br>(0.941)	0.239<br>(0.535)	-0.002<br>(0.996)
Rhizome length	-0.584<br>(0.224)	-0.059<br>(0.881)	-0.194<br>(0.616)	-0.223<br>(0.564)	-0.259<br>(0.501)	-0.362<br>(0.339)	-0.423<br>(0.257)	-0.482<br>(0.189)	-0.410<br>(0.273)	0.097<br>(0.803)
Root diameter	-0.439<br>(0.383)	-0.049<br>(0.901)	0.015<br>(0.970)	-0.068<br>(0.861)	-0.023<br>(0.954)	0.174<br>(0.655)	0.081<br>(0.836)	-0.149<br>(0.701)	-0.111<br>(0.777)	-0.446<br>(0.229)
Root length	-0.661<br>(0.153)	-0.234<br>(0.545)	0.355<br>(0.349)	0.165<br>(0.671)	0.290<br>(0.449)	0.550<br>(0.125)	0.420<br>(0.261)	-0.408<br>(0.276)	-0.300<br>(0.432)	-0.469<br>(0.203)
No. of rootlets	-0.290<br>(0.578)	0.255<br>(0.509)	0.236<br>(0.540)	0.224<br>(0.562)	0.290<br>(0.449)	0.226<br>(0.559)	0.272<br>(0.478)	-0.041<br>(0.918)	-0.146<br>(0.708)	-0.175<br>(0.652)
Total weight	-0.424<br>(0.403)	-0.478<br>(0.193)	-0.115<br>(0.769)	-0.272<br>(0.479)	-0.144<br>(0.712)	0.470<br>(0.202)	-0.275<br>(0.474)	-0.196<br>(0.614)	0.088<br>(0.821)	0.135<br>(0.729)
Aerial weight	-0.069<br>(0.897)	-0.495<br>(0.176)	-0.199<br>(0.607)	-0.305<br>(0.424)	-0.249<br>(0.519)	0.374<br>(0.321)	-0.219<br>(0.572)	0.072<br>(0.853)	0.380<br>(0.312)	-0.064<br>(0.869)

Root weight	-0.632<br>(0.178)	-0.018<br>(0.963)	0.061<br>(0.876)	-0.005<br>(0.989)	0.044<br>(0.911)	0.086<br>(0.826)	0.082<br>(0.834)	-0.357<br>(0.345)	-0.336<br>(0.377)	-0.438<br>(0.238)
Dry weight	-0.736<br>(0.096)	-0.005<br>(0.989)	0.125<br>(0.748)	0.043<br>(0.913)	0.114<br>(0.770)	0.153<br>(0.694)	0.094<br>(0.810)	-0.401<br>(0.284)	-0.384<br>(0.307)	-0.356<br>(0.347)

Correlation coefficient (r) written are significantly correlated between the variables compared. Negative values denote negative correlation and positive values denote positive correlation. Values in brackets means *P* value ($^{**}P<0.01$, $^{*}P<0.05$).
